# Anti cancer effects of curcumin: cycle of life and death

**DOI:** 10.1186/1747-1028-3-14

**Published:** 2008-10-03

**Authors:** Gaurisankar Sa, Tanya Das

**Affiliations:** 1 Division of Molecular Medicine, Bose Institute, P-1/12 CIT Scheme VII M, Kolkata, 700054, India

## Abstract

Increasing knowledge on the cell cycle deregulations in cancers has promoted the introduction of phytochemicals, which can either modulate signaling pathways leading to cell cycle regulation or directly alter cell cycle regulatory molecules, in cancer therapy. Most human malignancies are driven by chromosomal translocations or other genetic alterations that directly affect the function of critical cell cycle proteins such as cyclins as well as tumor suppressors, e.g., p53. In this respect, cell cycle regulation and its modulation by curcumin are gaining widespread attention in recent years. Extensive research has addressed the chemotherapeutic potential of curcumin (diferuloylmethane), a relatively non-toxic plant derived polyphenol. The mechanisms implicated are diverse and appear to involve a combination of cell signaling pathways at multiple levels. In the present review we discuss how alterations in the cell cycle control contribute to the malignant transformation and provide an overview of how curcumin targets cell cycle regulatory molecules to assert anti-proliferative and/or apoptotic effects in cancer cells. The purpose of the current article is to present an appraisal of the current level of knowledge regarding the potential of curcumin as an agent for the chemoprevention of cancer *via *an understanding of its mechanism of action at the level of cell cycle regulation. Taken together, this review seeks to summarize the unique properties of curcumin that may be exploited for successful clinical cancer prevention.

## Introduction

Cancers arise by an evolutionary process as somatic cells mutate and escape the restraints that normally rein in their untoward expansion. Consequently, multiple mechanisms have arisen to forestall uncontrolled cell division. Some of these are devices within the cell, such as those that limit cell-cycle progression, whereas others are social signals that prompt a cell to remain within its supportive microenvironment. In combination, these tumor-suppressing mechanisms are remarkably effective and can discriminate between neoplastic (abnormally growing) and normal cellular states and efficiently quell the former without suppressing the latter.

It is interesting to note that many, perhaps all, networks that drive cell proliferation harbor intrinsic growth-suppressive properties. Such innate inhibitory functions obscure any immediate selective advantage that mutations in such pathways might otherwise confer. Because no single pathway confers a net growth advantage, any proto-cancer cell acquiring any single oncogenic mutation is effectively trapped in an evolutionary cul-de-sac. By contrast in normal cells, coordinated extra-cellular cues activate multiple pathways in concert. In this way the inherent growth-suppressive activity of each pathway is gated by another, thereby unlocking the cell's proliferative potential. However, de-regulation of one or more of these activities may ultimately lead to cancer.

It is acknowledged that cancer results from the interaction of genetic susceptibility and environmental exposures. It is, therefore, not very unexpected that there are striking variations in the risk of different cancers by geographic area. These geographical variations indicate that there is clearly a strong environmental component to the risk differences. These patterns reflect in one hand prevalence of specific risk factors and on the other raise the possibility of presence of anti-cancer agents in the diet differentially depending on the food habit. Supporting both, migrant populations from high-risk parts of the world show a marked diminution in risk when they move to a lower risk area [1]. There is growing evidence that populations with greater reliance on fruits and vegetables in the diet experience a reduced risk for the major cancers [2]. The major classes of phytochemicals with disease-preventing functions are antioxidants, detoxifying agents and immunity-potentiating agents. Such dietary phytochemicals include curcumin (diferuloylmethane), a major naturally-occurring phenolic compound obtained from the rhizome of the plant *Curcuma longa*, which is used as a spice or yellow coloring agent for foods or drugs [3,4]. This phytochemical has long been known to have broad antioxidant properties [5]. Because curcumin can suppress cancer cell proliferation, induce apoptosis, inhibit angiogenesis, suppress the expression of anti-apoptotic proteins while protecting immune system of the tumor bearer – it may have untapped therapeutic value [3,6,7].

Recent studies using gene-array approach indicate that in any given type of cancer 300–500 normal genes have been altered/modified somehow to result in the cancerous phenotype. Although cancers are characterized by the deregulation of cell signaling pathways at multiple steps, most current anticancer therapies involve the modulation of a single target. The ineffectiveness, lack of safety, and high cost of mono-targeted therapies have led to a lack of faith in these approaches. As a result, many pharmaceutical companies are increasingly interested in developing multi-targeted therapies. Many plant-based products, however, accomplish multi-targeting naturally and, in addition, are inexpensive and safe compared to synthetic agents. However, because pharmaceutical companies are not usually able to secure intellectual property rights to plant-based products, the development of plant-based anticancer therapies has not been prioritized. Nonetheless, curcumin, a plant-based product, has shown significant promise against cancer and other inflammatory diseases.

In the present review we discuss how alterations in the cell cycle control contribute to the malignant transformation of normal cells and provide an overview of how curcumin targets cell cycle regulators to assert its anti-neoplastic effects. The purpose of the current article is to present an appraisal of the current level of knowledge regarding the potential of curcumin as an agent for the chemoprevention of cancer *via *an understanding of its mechanism of action at the level of cell cycle regulation.

### Cancer: cycle out of hand

Cell proliferation and cell death are such diametrically opposed cellular fates that how the two are linked and interdependent processes was a great surprise [8,9]. There is little mechanistic overlap between the machineries driving proliferation and apoptosis. Rather, the two processes are coupled at various levels through the individual molecular players responsible for orchestrating cell expansion. Importantly, the same players are often targets for oncogenic mutations, and in many instances, mutations that drive proliferation cooperate with those that uncouple proliferation from apoptosis during transformation and tumorigenesis [10,11]. But, although the phenomenon of oncogene-induced apoptosis is now generally accepted as an innate tumor-suppressive mechanism, we have only recently begun to glimpse the diversity and complexity of mechanisms by which oncogenic lesions engage the cell suicide machinery.

In normal cells there is a finely controlled balance between growth promoting and growth restraining signals such that proliferation occurs only when required. The balance tilts when increased cell numbers are required, e.g., during wound healing and during normal tissue turn over [12]. Proliferation and differentiation of cells during these processes occur in ordered manner and cease when no longer required. In tumor cells this process disrupts, continued cell proliferation occurs and loss of differentiation may be found. In addition, the normal process of programmed cell death that exists in normal cells may no longer operate [12]. In other words, a normal cell becomes malignant when the cellular proliferation is no longer under normal growth control. There are of course other characteristics that cancer cell may possess, such as angiogenesis, metastasis and suppression of apoptosis. But at the end the uncontrolled proliferation of the cell is at the heart of the disease. Therefore to understand cancer we need to transpire our knowledge on cell proliferation and its control.

**Figure 1:**
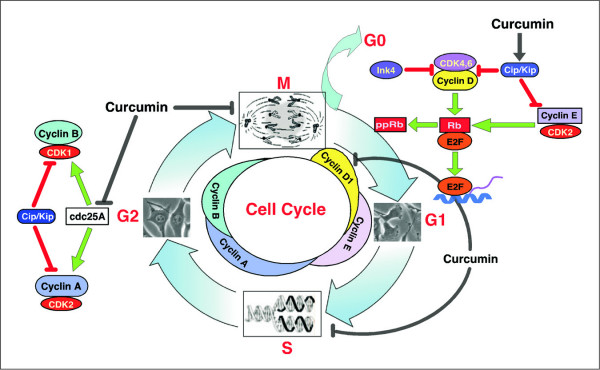
**The cell division cycle and its control**. The cell cycle is divided into four distinct phases (G1, S, G2, and M). The progression of a cell through the cell cycle is promoted by CDKs, which are positively and negatively regulated by cyclins and CKis, respectively. As shown, cyclin D isoforms interact with CDK4 and CDK6 to drive the progression of a cell through G1. Cyclin D/CDK4,6 complexes phosphorylate pRb, which releases E2F to transcribe genes necessary for cell cycle progression. The association of cyclin E with CDK2 is active at the G1-S transition and directs entry into S-phase. The INK4s bind and inhibit cyclin D-associated kinases (CDK4 and CDK6). The kinase inhibitor protein group of CKi, p21Cip1/Waf-1, p27Kip1, and p57Kip2, negatively regulate cyclin D/CDK4,6 and cyclin E/CDK2 complexes. S-phase progression is directed by the cyclinA/CDK2 complex, and the complex of cyclin A with Cdk1 is important in G2. CDK1/cyclin B is necessary for the entry into mitosis. Curcumin modulates CKis, CDK-cyclin and Rb-E2F complexes to render G1-arrest and alters CDK/cyclin B complex formation to block G2/M transition.

The process of replicating DNA and dividing a cell can be described as a series of coordinated events that compose a "cell division cycle". The mammalian cell cycle has been divided into a series of sequential phases. The G1, S, G2, and M phases are sequentially transitioned in response to growth factor or mitogenic stimulation (Figure 1). The DNA synthetic (S phase) and mitotic (M phase) phases are preceded by gap phases (G1, G2). Cell proliferation is tightly regulated by multiple interactions between molecules in normal cells. One molecular system senses growth-promoting conditions and sends a signal to a second set of molecules that actually regulates cell division. In addition, cells are equipped with signaling pathway that can sense unfavorable conditions for proliferation. This pathway antagonizes the proliferative signaling pathway and can directly block cell division [13-15]. Loss of integrity of these signaling pathways due to mutations can result in a hyper-proliferative state of cells, manifested as cancer [9,10]. Therefore, cancer is a disease of deregulated cell proliferation. It is becoming clear that many external signals including both those that stimulate growth, such as growth factors, and those that inhibit growth, such as DNA damaging agents, control cell proliferation through regulating the cell cycle. Thus, elucidating the machinery of cell cycle progression and its regulation by these signals is essential for understanding and controlling cell proliferation. Recent advances in our understanding of the cell cycle machinery in the last years have demonstrated that disruption of normal cell cycle control is frequently observed in human cancer [10,15].

### Cyclin-dependent pathway: the fuel of cell cycle

**Figure 2:**
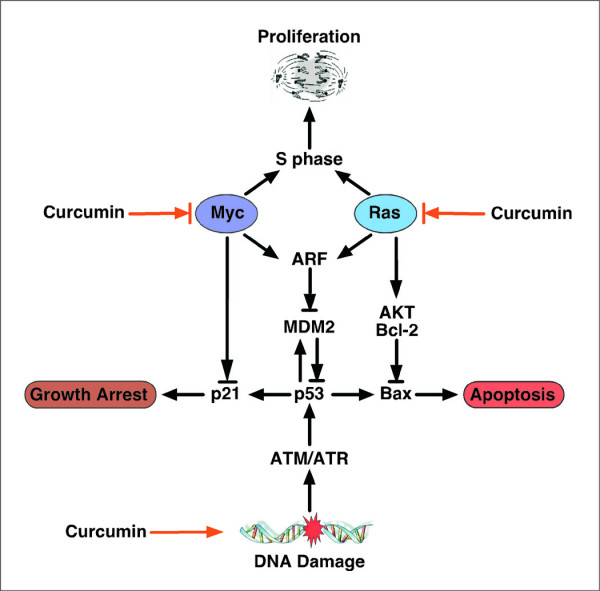
**The ARF-p53 circuit in tumour development and therapy**. Activation of Myc and Ras can force proliferation or trigger apoptosis. These oncogenic signals engage the tumor-suppressor network at many points, including through the ARF-p53 circuit shown here. Which components contribute most to tumor suppression depends on context. For example, Myc activates p53 to promote apoptosis while interfering with its ability to induce growth arrest by p21. Conversely, Ras activates p53 to promote growth arrest while suppressing apoptosis. This simplified view helps explain why, despite the potential of p53 to control several processes; apoptosis is primarily responsible for p53-mediated tumor suppression. DNA damage and oncogene signaling engage the tumor-suppressor network at different points and, as such, DNA-damage signaling relies more on p53 than on ARF to elicit an anti-proliferative response. Such a model explains why loss of ARF or p53 confers similar advantages during Myc-induced tumorigenesis but not following treatment with DNA-damaging drugs such as curcumin. Here, drug resistance is an unselected trait conferred by p53 mutations that provides a unique advantage as the tumor encounters a new environment (e.g., chemotherapy).

At least two types of cell cycle control mechanisms are recognized: a cascade of protein phosphorylations that relay a cell from one stage to the next and a set of checkpoints that monitor completion of critical events and delay progression to the next stage if necessary. The first type of control involves a highly regulated kinase family [13-15]. Kinase activation generally requires association with a second subunit that is transiently expressed at the appropriate period of the cell cycle; the periodic "cyclin" subunit associates with its partner "cyclin-dependent kinase" (CDK) to create an active complex with unique substrate specificity. Regulatory phosphorylation and dephosphorylation fine-tune the activity of CDK-cyclin complexes, ensuring well-delineated transitions between cell cycle stages. The orderly progression through G1 phase of the cell cycle is regulated by the sequential assembly and activation of three sets of cyclin-CDK complexes (Figure 2), the D cyclins (D1, D2 and D3) and CDK4 or CDK6, cyclin E and CDK2, cyclin A and CDK2 [14,15]. Genetic aberrations in the regulatory circuits that govern transit through the G1 phase of the cell cycle occur frequently in human cancer, and deregulated over-expression of cyclin D1 is one of the most commonly observed alterations that may serve as a drive oncogene through its cell-cycle regulating function [16]. In normal cells cyclin D1 expression is tightly regulated by mitogenic signals involving Ras pathway [17]. Increased cyclin D1 abundance occurs relatively early during tumorigenesis [18]. In most cancer types cyclin D1 over-expression results from induction by oncogenic signals, rather than a clonal somatic mutation or rearrangement in the *cyclin D1 *gene [19]. Tissue culture-based experiments evidenced cyclin D1 functions as a collaborative oncogene that enhances oncogenic transformation of other oncogenes (*i.e., Ras, Src, E1A*) [20,21]. Targeted expression of cyclin D1 or cyclin E induce mammary tumors [22,23]. The cyclin D- and E-dependent kinases contribute sequentially to the phosphorylation of the retinoblastoma gene susceptibility product (pRB), canceling its ability to repress E2F transcription factors and activating genes required for S phase entry [13,14].

Although the *RB-1 *gene was first identified through its role in a rare pediatric cancer, subsequent tumor studies have shown that this gene is sporadically mutated in a wide range of cancers [24]. In addition to direct mutation of the *RB-1 *gene, its encoded protein (pRB) is functionally inactivated in many tumor cells either by viral proteins that bind to pRB, or through changes in a regulatory pathway that controls the activity of pRB. Current mutation data indicates that nearly all tumor cells contain mutations or gene silencing events that effectively lead to inactivation of pRB. This establishes that pRB is necessary for restricting entry into the cell cycle and preventing cancer. This cyclin-CDK-mediated pathway leading to G1-S transition is known as "cyclin-dependent pathway". Regulation of G1-CDK activity is affected by their association with inhibitory proteins, called CDK inhibitors (CKi) [25]. So far, two families of CKi have been defined based on their structure and CDK targets: the Ink4 family and the Cip/Kip family [26]. The inhibitors of Ink4 family (p15^Ink4b^, p16^Ink4a^, p18^Ink4c ^and p19^Ink4d^) bind to monomeric Cdk4 and Cdk6 but not to Cdk2, thereby precluding the association of these Cdks to cyclins D [27]. Conversely, the members of Cip/Kip family, that include p21^Cip1/Waf-1^, p27^Kip1 ^and p57^Kip2^, all contain characteristic motifs at their N-terminal moieties that able them to bind both CDK and cyclins (Figure 1) [26,28]. It can thus be envisaged from the above discussion that any deregulation of this cyclin-dependent pathway can jeopardize the normal cell cycle progression and also that alteration of such deregulation can be one of the targets of cancer therapy. Therefore, the regulation of G1-S and G2-M transition could be an effective target to control the growth and proliferation of cancer cells, and facilitate their apoptotic death.

### p53: the master regulator

Besides "cyclin-dependent pathway", as a tumor suppressor, p53 has a central role in cell cycle regulation. However, this second type of cell cycle regulation, checkpoint control, is more supervisory. It is not an essential part of the cell cycle progression machinery. Cell cycle checkpoints sense flaws in critical events such as DNA replication and chromosome segregation [29]. When checkpoints are activated, for example, by under-replicated or damaged DNA, signals are relayed to the cell cycle-progression machinery. These signals cause a delay in cell cycle progression, until the danger of mutation has been averted. Because checkpoint function is not required in every cell cycle, the extent of checkpoint function is not as obvious as that of components integral to the process, such as CDKs. Researches conducted in the last two decades have firmly established the importance of p53 in mediating the cell cycle arrest that occurs following DNA damage, thus acting as a molecular "guardian of genome" (Figure 2) [8,30,31]. However, during the same time, the role of p53 in mediating apoptosis has become increasingly less clear, even as the number of putative pro-apoptotic proteins trans-activated by p53 has increased [8]. Numerous studies have analyzed the pattern of genes induced after p53 activation using global technologies such as SAGE, DNA array, Suppression Subtractive Hybridization or by cloning functional p53-binding sites. These studies emphasize the heterogeneity of the p53 response that is highly variable depending on the cell type, the nature and amount of DNA damage, the genetic background of the cells and the amount of p53 protein. Similarly unclear is how p53 makes a choice between cell-cycle arrest and apoptosis raising the possibility that p53 alone is not responsible for this crucial decision. An important function of p53 is to act as a transcription factor by binding to a p53-specific DNA consensus sequence in responsive genes, which would be expected to increase the synthesis of p21^Cip1 ^or Bax [8,30,31].

Up-regulation of p21^Cip1^/p21^Waf-1 ^results in the inhibition of cell cycle progression from G1 to S phase of cell cycle [32]. Interestingly, at Cip1, p53 pathway meets cyclin-dependent pathway. p21^Cip1 ^binds to cyclin-CDK complex, inhibits kinase activity and blocks cell cycle progression [32]. However, the underlying mechanism is still not yet fully revealed. Since the stabilization of another member of CKi family, p27^Kip1^, by phosphorylation prevents inhibition of Cdk/cyclin complexes in the ternary complex and blocks cell cycle progression [26,33,34], similar mechanism might be operative in case of p21^Cip1^. The available evidence suggests that Cip1-PCNA complexes block the role of PCNA as a DNA polymerase processivity factor in DNA replication, but not its role in DNA repair. Thus, Cip1 can act on cyclin-CDK complexes and PCNA to stop DNA replication. The removal of both *Cip1 *alleles from a cancerous cell line in culture that contained a wild-type *p53 *allele completely eliminated the DNA damage-induced G1 arrest in these cells, indicating that Cip1 is sufficient to enforce a G1 arrest in this experimental situation [35].

Another group of important regulators of apoptosis is the Bcl-2 family. These oncoproteins are classified into two groups: anti-apoptotic that inhibits apoptosis and pro-apoptotic that induces or accelerates it. The members form heterodimers to inactivate each other. The up-regulation of Bax expression and down-regulation of Bcl-2 have been demonstrated during apoptosis [32-36]. Interestingly, Bcl-2 over-expression renders cells resistant to apoptosis when it homodimerizes, whereas, up-regulation of Bax alters Bcl-2/Bax ratio in cellular microenvironment and cause release of cytochrome c from mitochondria into cytosol [37]. Cytochrome c then binds to Apaf-1 and activates caspase cascade, which is responsible for the later process of apoptosis [38]. Therefore, in one hand, deregulation of these cell cycle regulators leads to cancer and on the other any agent that can regulate these processes in cancer cells may have a role in tumor regression.

### Cell cycle and apoptosis: two sides of the same coin

The fundamental processes of progression through the cell cycle and of programmed cell death involve the complex interaction of several families of proteins in a systematic and coordinated manner. They are separate, distinct processes that are intimately related and together play an important role in the sensitivity of malignant cells to chemotherapy. The cell cycle is the mechanism by which cells divide. Apoptosis is an active, energy-dependent process in which the cell participates in its own destruction. The cell cycle and apoptosis are intimately related, as evidenced by the central role of p53, both in cell cycle arrest and in the induction of apoptosis. Another example of this intimate relation was demonstrated in human colon cancer cell lines that differ only in their p21 checkpoint status. Cells with wild-type p21, when irradiated with γ-radiation, underwent a cell cycle growth arrest followed by clonogenic survival, where as cells lacking p21, when irradiated with γ-radiation, did not undergo a cell cycle growth arrest and furthermore proceeded to apoptosis [39]. Cells that undergo a growth arrest may be protected from apoptosis and may therefore be ultimately resistant to the cytotoxic agent.

### Curcumin – the curry for cure: our hypothesis

Cell cycle progression is an important biological event having controlled regulation in normal cells, which almost universally becomes aberrant or deregulated in transformed and neoplastic cells. In this regard, targeting deregulated cell cycle progression and its modulation by various natural and synthetic agents are gaining widespread attention in recent years to control the unchecked growth and proliferation in cancer cells. In fact, a vast number of experimental studies convincingly show that many phytochemicals halt uncontrolled cell cycle progression in cancer cells. Among these phytochemicals, curcumin has been identified as one of the major natural anticancer agents exerting anti-neoplastic activity in various types of cancer cells. Here we hypothesize that curcumin asserts its anti-tumor activity in cancer cells by altering the de-regulated cell cycle *via *(a) cyclin-dependent, (b) p53-dependent and (c) p53-independent pathways.

### At the crossroads of alternative and main stream medicine

Turmeric has been used for thousands of years in Ayurvedic and traditional Chinese medicine. In modern times, curcumin, the yellow pigment of the spice turmeric, continues to be used as an alternative medicinal agent in many parts of South East Asia for the treatment of common ailments such as stomachic upset, flatulence, jaundice, arthritis, sprains, wounds and skin infections among many others. Curcumin and turmeric products have been characterized as safe by health authorities such as the Food and Drug Administration (FDA) in United States of America, Food and Agriculture Organization/World Health Organization (FAO/WHO). Curcumin has entered scientific clinical trials at the phase I and II clinical trial level only in the last 10–15 years. A phase III study of gemcitabine, curcumin and celecoxib is due to open to recruitment at the Tel-Aviv Sourasky Medical Center for patients with metastatic colorectal cancer [40].

### Why curcumin?

**Figure 3:**
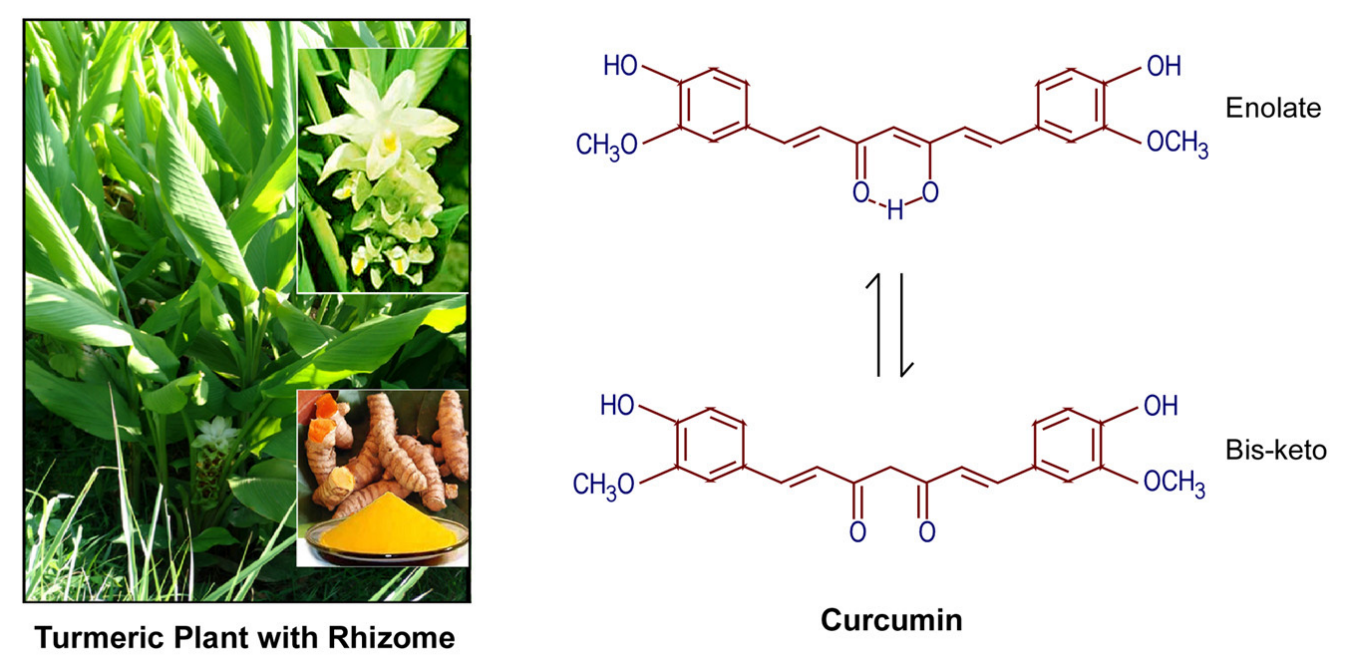
***Curcuma longa *Plant and chemical structure of curcumin, the active ingradient of rhizome termeric**. The tautomerism of curcumin is demonstrated under different physiological conditions. Under acidic and neutral conditions, the *bis-keto *form (bottom) is more predominant than the *enolate *form.

Curcumin is a component of turmeric; the yellow spice derived from the roots (rhizomes) of the plant *Curcuma longa*. *Curcuma longa *is a short-stemmed perennial, which grows to about 100 cm in height. It has curved leaves and oblong, ovate or cylindrical rhizomes (Figure 3). *Curcuma longa *grows naturally throughout the Indian subcontinent and in tropical countries, particularly South East Asia. A traditional remedy in "Ayurvedic medicine" and ancient Indian healing system that dates back over 5,000 years, turmeric has been used through the ages as an "herbal aspirin" and "herbal cortisone" to relieve discomfort and inflammation associated with an extraordinary spectrum of infectious and autoimmune diseases [4].

Curcumin, chemically it is known as diferuloylmethane (C_21_H_20_O_6_), has been the subject of hundreds of published papers over the past three decades, studying its antioxidant, anti-toxic, anti-inflammatory, cancer chemopreventive and potentially chemotherapeutic properties [3,4,41-44]. The pharmacology and putative anti-cancer properties of curcumin have been the subject of several review articles published since 1991, which predate a number of clinical studies of curcumin which have been completed and published within the last few years [45]. But these properties do not prove the superiority of this phytochemical over other chemotherapeutic agents that also induced apoptosis successfully in cancer cells.

Majority of chemotherapeutic agents, including those isolated from plants (such as taxol or vincristin etc.) not only induce cancer cell apoptosis but also severely damage the normal cells of the host, the effects being particularly severe in case of the immune system [46]. On the contrary, curcumin is a part of our daily food habit and its use in large quantities from ancient time has already proved that it is a safe product [4]. In fact, since curcumin preferably induces apoptosis in highly proliferating cells, death is much more pronounced in tumor cells than normal ones [47]. Report from our laboratory has shown that anticancer dose of curcumin arrests non-malignant cells in G0 phase reversibly but does not induce apoptosis in them [6]. Further studies revealed that this phytochemical protects T cells of the cancer bearer from cancer as well as chemotherapeutic agent-induced apoptosis [7,47]. The basis of this differential regulation may be attributed to its differential effects on normal and neoplastic cell cycles since deregulation of some components of cell cycle regulatory machinery can drive uncontrolled proliferation and hence neoplastic transformations.

The broad biological activity of this phytochemical, including antioxidant and metabolic effect, influences upon key signal transduction pathways of cell cycle and effectiveness in animal model systems have fostered development of translational, and clinical research programs. In pilot clinical studies in India, Taiwan, USA and UK, curcumin has been associated with regression of pre-malignant lesions of the bladder, soft palate, GI tract, cervix, and skin, and with treatment responses in established malignancy [48-52]. Doses up to 8–10 g could be administered daily to patients with pre-malignant lesions for 3 months without overt toxicity [48-50]. It cannot be assumed that diet-derived agents will be innocuous when administered as pharmaceutical formulations at doses likely to exceed those consumed in the dietary matrix. Anecdotal reports suggest that dietary consumption of curcumin up to 150 mg/day is not associated with any adverse effects in humans [44]. The epidemiological data interestingly suggest that it may be reason for the lower rate of colorectal cancer in these countries than in "developed" countries [1,2]. The preclinical data in human subjects suggest that a daily dose of 3.6 g curcumin achieves measurable levels in colorectal tissue. Efficient first-pass and some degree of intestinal metabolism of curcumin, particularly glucuronidation and sulphation, may explain its lesser systemic availability when administered *via *oral route [53]. So, gastrointestinal tract could represent a preferential chemoprevention target because of its greater exposure to unmetabolized bioactive curcumin from diet than other tissues. All these information not only suggest that curcumin has enormous potential in the prevention and therapy of cancer but also well justify the utility of using curcumin as an anti-tumor agent.

### To arrest or to kill – two weapons of curcumin

It is now apparent that many of the phytochemicals preferentially inhibit the growth of tumor cells by inducing cell cycle arrest or apoptosis (Figure 2). The anti-tumor effect of curcumin has also been attributed in part to the suppression of cell proliferation, reduction of tumor load and induction of apoptosis in various cancer models both *in vitro *and *in vivo *[6,44,48,49,54-57]. Curcumin inhibits multiple levels within transcriptional network to restrict cell proliferation. It induces p53-dependent apoptosis in various cancers of colon, breast, bladder, neuron, lung, ovary etc., although both p53-dependent and -independent G2/M phase arrest by curcumin has been observed in colorectal cancer cells [6,48,49,57-61]. Curcumin promotes caspase-3-mediated cleavage of β-catenin, decreases β-catenin/Tcf-Lef transactivation capacity for c-Myc and cyclin D1 [62]. It also activates caspase-7 and caspase-9 and induces polyadenosine-5'-diphosphate-ribose polymerase cleavage through the down-regulation of NFκB in multiple myeloma cells [63]. Furthermore, curcumin inhibits EGFR activation [64], Src activity [65] and inhibits activity of some nuclear receptors [66]. Curcumin inhibitory effects upon Cox-2 and cyclin D1, mediated through NF-κB, also restrict tumor cell growth [62,67]. Induction of G2/M arrest and inhibition of Cox-2 activity by curcumin in human bladder cancer cells has also been reported [58]. It induces colon cancer cell apoptosis by JNK-dependent sustained phosphorylation of c-Jun [68] and enhances TNF-α-induced prostate cancer cell apoptosis [70]. In fact, curcumin induces apoptosis in both androgen-dependent and androgen-independent prostate cancer cells [70]. On the other hand, in breast carcinoma cells, it inhibits telomerase activity through human telomerase reverse-transcritpase [71]. In Bcr-Abl-expressing cells, G2/M cell cycle arrest, together with increased mitotic index and cellular as well as nuclear morphology resembling those described for mitotic catastrophe, was observed and preceded caspase-3 activation and DNA fragmentation leading to apoptosis [72]. Curcumin arrested cell growth at the G2/M phase and induced apoptosis in human melanoma cells by inhibiting NFκB activation and thus depletion of endogenous nitric oxide [73]. However, in mantle cell lymphoma curcumin has been found to induce G1/S arrest and apoptosis [74]. In T cell leukemia curcumin induced growth-arrest and apoptosis in association with the inhibition of constitutively active Jak-Stat pathway and NFκB [75,76]. Holy [77] reported disruption of mitotic spindle structure and induction of micronucleation in human breast cancer cells by this yellow pigment. Besides arresting growth or inducing apoptosis, curcumin also enhances differentiation by targeting PI3K-Akt pathway, Src-mediated signaling and PPAR [64,65,78]. This action of curcumin promotes cells exit from cycle. All these reports indicate that curcumin might be asserting its anti-cancer effect by modulating cancer cell cycle regulatory machineries.

### Curcumin: the manipulator of cyclin pathway

It is clear that curcumin spares normal cell from apoptotic induction making it a relatively safe anti-cancer agent. The question thus arises that what confers this selectivity. In an attempt to understand the basic mechanisms of carcinogenesis, it was found that, in slowly-proliferating non-malignant cells, Ras activity is stimulated to high level at G1 phase upon mitogenic challenge and leads to cyclin D1 elevation during mid to late G1 phase [13-16]. Interestingly, we found that this pattern, upon which most models of cell cycle regulation are based, does not apply to actively proliferating cancer cells. In fact, in these rapidly cycling cells, oncogenic Ras is active throughout the cell cycle during exponential growth and induces high levels of cyclin D1 expression in G2 phase that continues through mitosis to G1 phase bypassing G0 phase, a phase that regulates uncontrolled proliferation [79-81]. These results not only demonstrated that the critical signaling events upon which cell cycle progression depends take place during G1 phase in normal cells, but during G2 phase in actively growing cancer cells but also that G2 phase of cell cycle plays a critical role in controlling hyper-proliferative status of cancer cell and is thus susceptible to successful anti-cancer drug therapy.

**Figure 4:**
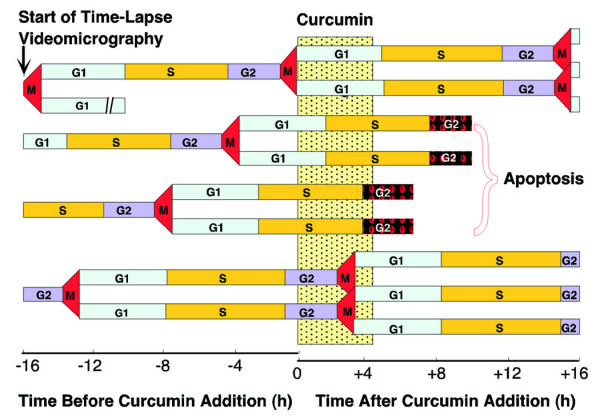
**Time-lapse determination of approximate cell cycle position of curcumin-induced apoptosis**. Time-lapse video-micrography was employed to monitor curcumin-induced apoptosis of breast cancer cells. Age of each cell was analyzed from a time-lapse analysis before curcumin addition. The occurrence and the time of apoptosis after curcumin addition were determined from a time-lapse analysis after addition.

With elegant time-lapse video-micrography and quantitative imaging approach our works with breast malignant cells and adjacent non-malignant cells indicate that curcumin did not alter the cell cycle progression of carcinoma cells, although it induced apoptosis in the same at G2 phase of cell cycle (Figure 4) while reversibly blocking non-malignant cell cycle progression without apoptosis [6]. An interesting finding in this study was that curcumin appeared to be sparing the normal epithelial cells by arresting them at the G0 phase of the cell cycle *via *down-regulation of cyclin D1 and its related protein kinases or up-regulation of the inhibitory protein. The experiments with cyclin D1-deregulated cells showed that curcumin did not alter cyclin D1 expression level in cancer cells, but in normal cells, where cyclin D1 expression is tightly regulated by mitogenic signaling, its expression is inhibited by curcumin. This inability of curcumin to inhibit cyclin D1 expression in cyclin D1-deregulated cells may serve as the basis for differential regulation of cancerous and normal cells. In addition, curcumin was found to inhibit the association of cyclin D1 with CDK4/CDK6 or phosphorylation of pRb in some cancer cells where the expression of cyclin D1 is not deregulated and thus arrest them at G0/G1 phase (Figure 1) [82,83]. This yellow pigment has been shown to inhibit neoplastic cell proliferation by decreasing Cdk1 kinase activity and arresting cells at G2/M check point [81]. Ectopically over-expression of cyclin D1 renders susceptibility of these cells towards curcumin toxicity [6]. These results may well explain why in cancer cells, despite up-regulation of p53 and increase in Cip1 level, there was no cell cycle arrest. In fact, the level of cyclin D1 is very high in these cells and remained unchanged upon curcumin treatment. Thus, the amount of Cip1, as up regulated by curcumin, was still not sufficient to overpower cyclin D1 and to stop cell cycle progression. On the other hand, in non-malignant cells, the level of Cip1 increased dramatically with parallel down-regulation of cyclin D1, thereby making the ratio of Cip1 to cyclin D1 > 1 and this might be one of the causes of cell cycle arrest without apoptosis [6]. The above discussion not only relates curcumin activity with cell cycle regulation but also explains the mechanism underlying the differential effect of this phytochemical in normal and malignant cells.

### Curcumin regulating "guardian of genome"

The tumor suppressor gene *p53*, acknowledged as the *"guardian of genome"*, is situated at the crossroads of a network of signaling pathways that are essential for cell growth regulation and apoptosis [30-35]. In normal unstressed cells, these upstream pathways predominantly include the binding by proteins such as Mdm2 that promote p53 degradation *via *the ubiquitin-26S proteasome pathway [32]. COP9 signalosome (CNS)-specific phosphorylation targets p53 to ubiquitin-26S proteasome-dependent degradation. Curcumin has been found to inhibit CSN and block Mdm2- and E6-dependent p53 degradation [84]. Furthermore, in basal cell carcinoma, curcumin promotes *de novo *synthesis of p53 protein or some other proteins for stabilization of p53, and hence enhances its nuclear translocation to transactivate Cip1 and Gadd45 indicating that p53-associated signaling pathway is critically involved in curcumin-mediated apoptotic cell death [56]. With time-lapse video-micrography and quantitative imaging approach we have demonstrated that in deregulated cells, curcumin induces p53 dramatically at G2 phase of cell cycle and enhances p53 DNA-binding activity resulting in apoptosis at G2 phase (Figure 4) [6,47]. On the other hand, curcumin increases p53 expression to a lower extent throughout the cell cycle in non-malignant cells [6]. In these cells, curcumin reversibly up-regulates Cip1 expressions and inactivates pRB and thus arrests them in G0 phase of cell cycle. Therefore, these cells escape from curcumin-induced apoptosis at G2 phase. Works from other laboratories also suggest that curcumin induces p53 expression in colon, breast, and other cancer cells [57-61]. Reports from our laboratory as well as from other laboratories suggest that curcumin predominantly acts in a p53-dependent manner as careful analysis of the effect of curcumin in various cells expressing wild-type or mutated p53 as well as cells transfected with dominant-negative p53, revealed that the cells expressing high levels of wild-type p53 were more sensitive to curcumin toxicity. On the other hand, p53-knock-out as well as p53-mutated cells also showed toxicity, although the apoptotic-index is lower [6,42,47].

Search for downstream of p53 revealed that in mammary epithelial carcinoma and colon adenocarcinoma cells curcumin could increase the expression of the pro-apoptotic protein Bax and decrease the anti-apoptotic protein Bcl-2/Bcl-xL through the phosphorylation at Ser15 and activation of p53 [6,85]. Our results also revealed curcumin-induced G2/M arrest and apoptosis of mammary epithelial carcinoma cells *via *p53-mediated Bax activation [6,47]. On the other hand, c-Abl, a non-receptor tyrosine kinase, has been reported to play an important role in curcumin-induced cell death through activation of JNK and induction of p53 [86].

All these reports indicate that curcumin can induce cancer cell killing predominantly *via *p53-mediated pathway, p53 not only controls apoptotic pathways but also acts as a key cell cycle regulatory protein as it can trans-activate cell cycle inhibitors like Cip1 on the event of DNA damage during proliferation and when the damage is irreparable it induces apoptosis by inducing the expression of pro-apoptotic proteins like Bax (Figure 2). So far our discussion thus clearly indicates the involvement of the *guardian of genome*, p53, in curcumin-induced cancer cell apoptosis *via *cell cycle regulation.

### p53-independent pathways and curcumin

**Figure 5:**
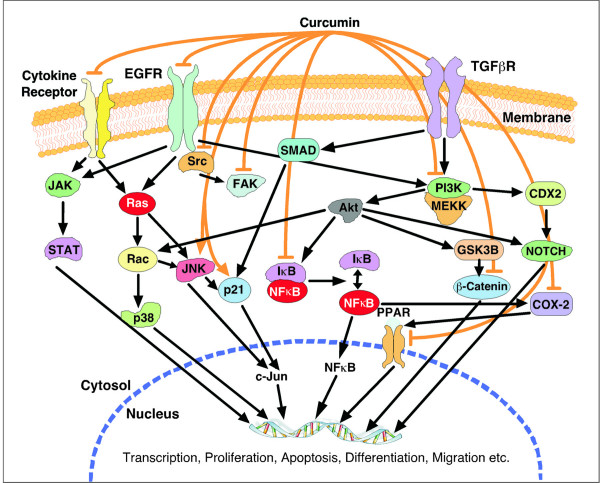
**Oncogenic signaling targets many levels curcumin**. Curcumin enhances apoptotic death, inhibits deregulated cellular proliferation, dedifferentiation and progression towards the neoplastic phenotype by altering key signaling molecules required for cell cycle progression. Such a network organization allows the cell to sense many aspects of the intracellular and extra-cellular milieu, yet ensures that cell death proceeds efficiently once activated. Excessive oncogenic signaling is coupled to apoptosis by a complex mechanism that targets key control points in the pathways. Blunt-head lines indicate that these molecules can be down-regulated by curcumin, where as arrow-head lines indicate that these molecules are often up-regulated by curcumin.

It is evident that curcumin can induce selective cancer cell killing in a p53-dependent manner, but impaired p53 expression or activity is associated with a variety of neoplastic transformations. Increasing reports are indicating that curcumin can block cell cycle progression or even apoptosis in a p53-independent manner as well, especially in the cells that lack functional p53 [83]. Curcumin induces apoptosis in p53-null lung cancer cells [61]. It induces melanoma cell apoptosis by activating caspase-8 and caspase-3 *via *Fas receptor aggregation in a FasL-independent manner, blocks NFκB cell survival pathway and suppresses the apoptotic inhibitor XIAP [87]. Curcumin inhibits cellular isopeptidases, and cause cell death independently of p53 in isogenic pairs of RKO and HCT 116 cells with differential p53 status [88]. It enhances the chemotherapy-induced cytotoxicity in p53-null prostate cancer cell line PC-3, *via *up-regulation of Cip1 and C/EBPβ expressions and suppression of NFκB activation [89]. It also induces apoptosis in multiple myloma cells by inhibiting IKK and NFκB activity [64]. Study indicates that curcumin down regulates NFκB and AP-1 activity in androgen-dependent and -independent prostate cancer cell lines [70]. Curcumin is a potent inhibitor of protein kinase C (PKC), EGF (epidermal growth factor)-receptor tyrosine kinase and IκB kinase. Subsequently, curcumin inhibits the oncogenes including *c-jun, c-fos, c-myc, NIK, MAPKs, ELK, PI3K, Akt, CDKs *and *iNOS *[63,90]. In contrast to the mentioned reports, studies by Collet *et al. *shows that curcumin induces JNK-dependent apoptosis of colon cancer cells and it can induce JNK-dependent sustained phosphorylation of c-jun and stimulation of AP-1 transcriptional activity [68]. The oxidized form of cancer chemopreventive agent curcumin can inactivate PKC by oxidizing the vicinal thiols present within the catalytic domain of the enzyme [90]. Recent studies indicated that proteasome-mediated degradation of cell proteins play a pivotal role in the regulation of several basic cellular processes including differentiation, proliferation, cell cycling, and apoptosis. It has also been demonstrated that curcumin-induced apoptosis is mediated through the impairment of ubiquitin-proteasome pathway [90]. All these reports suggests that curcumin can induce apoptosis or block cell cycle progression in a variety of cancer cell lines, predominantly *via *p53-dependent pathways, but it can also act in a p53-independent manner (Figure 5).

### Other functions of curcumin

Curcumin inhibits angiogenesis directly and via regulation of angiogenic growth factors like vascular endothelial growth factor, basic fibroblast growth factor and epidermal growth factor, as well as the genes like angiopoietin 1 and 2, hypoxia-inducible factor-1, heme oxygenase-1, and the transcriptional factors like NF-κB [40]. Inhibition of angiogenic growth factor production and metalloproteinase generation, both integral to the formation of new vasculature, has also been influenced by curcumin in non-malignant and malignant cells growth [91,92]. Similar to the inhibition of angiogenic factors, curcumin has been shown to regulate proteins related to cell-cell adhesion, such as β-catenin, E-cadherin and APC and to inhibit the production of cytokines relevant to tumor growth, e.g. tumour necrosis factor-α (TNF-α) and interleukin-1 [93,94]. Additionally, curcumin has been shown to reduce the expression of membrane surface molecules such as intracellular adhesion molecule-1, vascular cell adhesion molecule-1 and E-selectin and matrix metaloproteases those play important roles in cellular adhesion and metastasis [3,95].

Curcumin has also been shown to quench reactive oxygen species and scavenge superoxide anion radicals and hydroxyl radicals and strongly inhibits nitric oxide (NO) production by down-regulating inducible nitric oxide synthase gene expression [96,97]. Curcumin inhibits of phase I enzymes systems consist of cytochrome P450 isoforms, the P450 reductase, the cytochrome b5 and the epoxide hydrolase and protect from the toxic effects of chemicals and carcinogens [60]. On the other hand curcumin induces phase II enzymes (glutathione S-transferases and epoxide hydrolase), which play a protective role by eliminating toxic substances and oxidants and conferring benefit in the prevention of the early stages of carcinogenesis [98].

Curcumin can act as a potent immunomodulatory agent that can modulate the activation of T cells, B cells, macrophages, neutrophils, natural killer cells, and dendritic cells. Curcumin can also down-regulate the expression of various pro-inflammatory cytokines including TNF, IL-1, IL-2, IL-6, IL-8, IL-12, and chemokines, most likely through inactivation of the transcription factor NF-κB [99]. Interestingly, however, curcumin at low doses can also enhance antibody responses. Curcumin has been shown to activate host macrophages and natural killer (NK) cells and modulate of lymphocyte-mediated functions [100]. Studies from our laboratory showed that curcumin neutralized tumor-induced oxidative stress, restored NF-kB activity, and inhibited TNF-α production, thereby minimizing tumor-induced T-cell apoptosis [7]. Further work suggests that curcumin helps in T cell survival both in primary and effecter immune compartments of tumor-bearing hosts by normalizing perturbed of Jak-3/Stat-5 activity via restoration of IL2-receptor γc chain expression [101]. Curcumin was found to prevent tumor-induced loss of T-effector cells, reverse type-2 cytokine bias and blocks T-regulatory cell augmentation in tumor-bearing hosts via down-regulation of TGF-β in cancer cells (unpublished data). From all these observations it is suggested that curcumin may be used alone or can be combined with classical anti-tumor drugs so as to sustain the immune capacity of the host, which can be affected by the disease or the treatment or may be the both.

### Curcumin – a multiple edged sword

Above discussions on the broad biological activity of this phytochemical prove our hypothesis that curcumin asserts its anti-tumor activity in cancer cells by altering the deregulated cell cycle *via *(a) cyclin-dependent, (b) p53-dependent and (c) p53-independent pathways. Such influences of curcumin upon key signal transduction pathways of cell cycle and effectiveness in animal model systems have qualified it as a *multiple edged sword *in combating the deadly disease – cancer. Given that disruption of cell cycle plays a crucial role in cancer progression, its modulation by curcumin seems to be a logical approach in controlling carcinogenesis. Most of the plant products with anticancer activity act as strong antioxidants and some of them are effective modulators of protein kinases/phosphatases that are associated with cell cycle regulation. Many of these phytochemicals are either part of the human diet or consumed as dietary supplement, and do not show adverse health effects even at large doses. Due to failure of conventional chemotherapy in advance stages of cancer and its enormous adverse effects, cancer chemoprevention by this phytochemical in a defined molecular target approach will play an important role in future in reducing cancer incidence as well as the number of deaths caused by this disease.

### Prospects for the future

Previous seminal work, summarized above has demonstrated curcumin inhibition of key molecular mechanisms of tumorigenesis. Effects have been shown of common signaling intermediates that influence the tumor phenotype. Major advances in the understanding of cell cycle regulation mechanisms provided a better knowledge of the molecular interactions involved in human cancer. Moreover, the components of the cell cycle are probably involved in other non-cancerous diseases and their role must be defined. Further mechanistic work however, is required to investigate curcumin effects on switches that connect common effector pathways that regulate cell behavior, phenotype alteration and cell death or lineage commitment. Human intervention studies of curcumin, whether alone or in combination, are indicated against intermediate biomarkers and morphological stages of gastrointestinal tumorigenesis. Curcumin could thus provide a useful component of dietary or pharmacological treatment aimed at reduction of the incidence of and mortality from cancer.

## Competing interests

The authors declare that they have no competing interests.

## Authors' contributions

GS and TD contributed to the discussion and preparation of this manuscript. Both the authors read and approved the final manuscript.
